# A New Tool for Extracting Static and Dynamic Parameters from [^18^F]F-DOPA PET/CT in Pediatric Gliomas

**DOI:** 10.3390/jcm13206252

**Published:** 2024-10-19

**Authors:** Michele Mureddu, Thomas Funck, Giovanni Morana, Andrea Rossi, Antonia Ramaglia, Claudia Milanaccio, Antonio Verrico, Gianluca Bottoni, Francesco Fiz, Arnoldo Piccardo, Marco Massimo Fato, Rosella Trò

**Affiliations:** 1 Department of Informatics, Bioengineering, Robotics and System Engineering (DIBRIS), University of Genoa, 16145 Genoa, Italy; michele.mureddu@edu.unige.it (M.M.); marco.fato@unige.it (M.M.F.); rosella.tro@edu.unige.it (R.T.); 2 Child Mind Institute, New York, NY 10022, USA; thomas.funck@childmind.org; 3 Department of Neurosciences, University of Turin, 10126 Turin, Italy; giovanni.morana@unito.it; 4 NeuroRadiology Unit, IRCCS Institute Giannina Gaslini, 16147 Genoa, Italy; andrearossi@gaslini.org (A.R.); antoniaramaglia@gaslini.org (A.R.); 5 Neuro-Oncology Unit, IRCCS Institute Giannina Gaslini, 16147 Genoa, Italy; claudiamilanaccio@gaslini.org (C.M.); antonioverrico@gaslini.org (A.V.); 6 Nuclear Medicine Unit, Ente Ospedaliero Ospedali Galliera, 16128 Genoa, Italy; gianluca.bottoni@galliera.it (G.B.); francesco.fiz@galliera.it (F.F.)

**Keywords:** automatic pipeline, brain gliomas, [^18^F]F-DOPA PET imaging, FLAIR MRI, pediatric gliomas

## Abstract

**Background/Objectives**: PET imaging with [^18^F]F-DOPA has demonstrated high potential for the evaluation and management of pediatric brain gliomas. Manual extraction of PET parameters is time-consuming, lacks reproducibility, and varies with operator experience. **Methods**: In this study, we tested whether a semi-automated image processing framework could overcome these limitations. Pediatric patients with available static and/or dynamic [^18^F]F-DOPA PET studies were evaluated retrospectively. We developed a Python software to automate clinical index calculations, including preprocessing to delineate tumor volumes from structural MRI, accounting for lesions with low [^18^F]F-DOPA uptake. A total of 73 subjects with treatment-naïve low- and high-grade gliomas, who underwent brain MRI within two weeks of [^18^F]F-DOPA PET, were included and analyzed. Static analysis was conducted on all subjects, while dynamic analysis was performed on 32 patients. **Results**: For 68 subjects, the Intraclass Correlation Coefficient for T/S between manual and ground truth segmentation was 0.91. Using our tool, ICC improved to 0.94. Our method demonstrated good reproducibility in extracting static tumor-to-striatum ratio (*p* = 0.357); however, significant differences were observed in tumor slope (*p* < 0.05). No significant differences were found in time-to-peak (*p* = 0.167) and striatum slope (*p* = 0.36). **Conclusions**: Our framework aids in analyzing [^18^F]F-DOPA PET images of pediatric brain tumors by automating clinical score extraction, simplifying segmentation and Time Activity Curve extraction, reducing user variability, and enhancing reproducibility.

## 1. Introduction

Gliomas are the most common central nervous system (CNS) neoplasms in childhood, accounting for 35% of pediatric CNS tumors [1]. They encompass a wide range of entities (including astrocytic and mixed neuronal-glial tumors), exhibiting different growth patterns (from diffuse to circumscribed), and comprising both high- and low-grade malignancies [1]. Gliomas present different characteristics between pediatric and adult age, such as genetic mutations and response to therapy [2]. In the assessment of pediatric brain gliomas, PET imaging employing amino acid radionuclides has demonstrated the ability to address limitations of (i) conventional magnetic resonance imaging (MRI) [2] and (ii) the restricted contrast associated with the use of the radiopharmaceutical 18F-fluorodeoxyglucose ([^18^F]-FDG) in neuro-oncology [3,4]. Furthermore, 18F-labeled tracers are becoming more prevalent due to their extended half-life, in contrast to 11C tracers [5]. Among these radionuclides, 18F-dihydroxyphenylalanine (DOPA) has emerged as particularly notable because its uptake significantly correlates with tumor grading [6] and prognosis [7] in patients with infiltrating gliomas. Additionally, [^18^F]F-DOPA PET demonstrated a potential role to non-invasively determine the H3K27M mutation status in diffuse midline gliomas (DMG), such as diffuse intrinsic pontine gliomas (DIPG), which are malignant brainstem gliomas where biopsy is not routinely performed since diagnosis can still be made based on clinical and imaging features alone [8,9,10]. In clinical practice, [^18^F]F-DOPA PET analysis of pediatric gliomas is based on standard parameters such as tumor-to-striatum (T/S) and tumor-to-background tissue (T/N) ratios. However, these parameters may show limitations for certain lesion types, and in recent years, the use of dynamic parameters (i.e., the shape of the time activity curve and the steepness of the uptake curve) has increased to overcome these limitations [10,11]. Of note, in 2022 Fiz et al. [12] conducted a pioneering study of static and dynamic [^18^F]F-DOPA PET parameters in 15 children with brain gliomas. They highlighted the correlation of the T/S ratio with outcomes and observed that time-activity curve (TAC) patterns best correlated with OS and progression-free survival (PFS). Two distinct patterns were identified: an accumulated trend in low-grade neoplasms and a plateau pattern in aggressive gliomas, consistent with the guidelines in [13]. Despite these significant research advancements, highlighting the diagnostic and prognostic value of [^18^F]F-DOPA PET-derived parameters, clinical practice still faces challenges in accurately and reproducibly measuring these markers. Nuclear medicine physicians typically measure quantitative markers from PET scans with limited computer assistance. For instance, they delineate a circular ROI centered on the maximum tumor and striatum uptake to compute maximum T/S. When the lesion uptake resembles that of healthy brain tissue, clinicians manually place a ROI on the area of signal abnormality in the T2-weighted MRI scan and transfer it to the coregistered PET scan. The normal uptake region (N) is traditionally delineated by positioning a sphere on the contralateral semi-oval center, including gray and white matter [14,15]. This approach is also applied to TAC extraction, where segmentation is particularly challenging due to the limited anatomical information within the dynamic PET scans. This working method is time consuming and has low reproducibility, so it is essential to define an automatic workflow that is less dependent on the manual definition of ROI or VOI. Our work aims to implement a semi-automated image processing framework for [^18^F]F-DOPA PET, integrating it into the clinical workflow of neuro-oncology. This pipeline is designed to extract both static and dynamic parameters to support nuclear medicine specialists in assessing pediatric brain tumors by accelerating analysis time and improving the accuracy of PET-derived parameters to overcome the inherent drawbacks of manual analysis.

## 2. Materials and Methods

### 2.1. Patient Population

A retrospective evaluation was conducted on all consecutive pediatric patients (aged younger than 18 years on diagnosis) referred to our institutions between 2011 and 2024 for newly diagnosed treatment-naïve (except biopsy) brain gliomas who had undergone conventional MRI and [^18^F]F-DOPA PET (including static and/or dynamic acquisition) within 2 weeks of each other. Tumor grade was determined according to the 2021 WHO Classification of Tumors of the Central Nervous System. Exclusion criteria were poor image quality due to artifacts (e.g., movement, altered distribution due to venous extravasation, etc.), multiple active malignancies, unavailability of MRI images, or lack of histological confirmation. Patients’ characteristics are reported in Table 1 following nomenclature reported in [16].

### 2.2. Image Protocol

[^18^F]F-DOPA PET studies were executed with a Discovery ST PET/CT or with a Discovery MI PET/CT system (GE Healthcare, Milwaukee, WI, USA). Both static and dynamic acquisitions were included in the analysis. [^18^F]F-DOPA was administered as a bolus injection. PET scans were acquired using a ninety-second emission scan per bed position from the lower extremities to the vertex. Raw PET data were reconstructed using an OSEM algorithm (four iterations, eight subsets, and a 5 mm filter), and the reconstructed voxel size was 2.027 × 2.036 × 2.036 mm with a 256 × 256 matrix. Low-dose CT was used for both attenuation correction and topographic localization. MRI studies were performed on 1.5T (Intera Achieva, Philips, Best, The Netherlands) or 3T (Ingenia Cx, Philips, Best, The Netherlands) scanners. All patients underwent routine clinical MRI examinations including axial fluid attenuation inversion recovery (FLAIR), T2-weighted images, and pre- and post-contrast (0.1 mmol/kg, macrocyclic ionic agent) T1-weighted images.

### 2.3. Implementation

**Figure 1:**
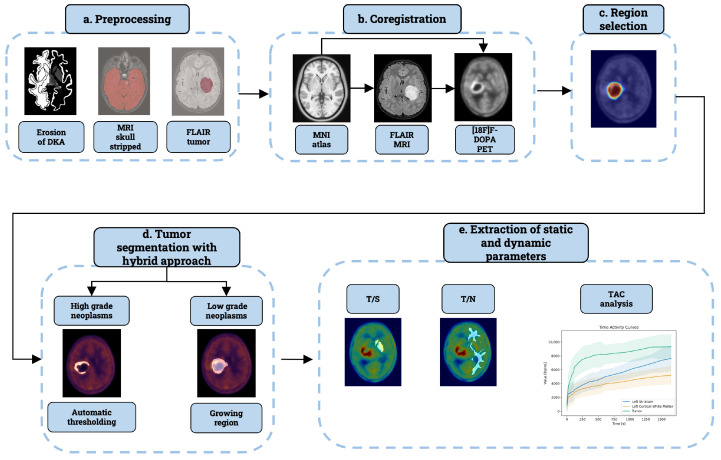
Image analysis framework is composed of 5 interconnected modules. (**a**) The first module consists of pre-processing, that is, (i) the erosion of reference regions to dissipate the impact of partial volume effect, (ii) FLAIR MRI skull stripping to eliminate the high level of uptake in non-encephalic regions, and (iii) FLAIR tumor delineation with advanced drawing tool of MRIcroGL. All the outcomes of this step are used as input for subsequent pipeline steps. (**b**) The second module is represented by the registration of different imaging modalities in PET coordinate space. (**c**) Reference regions are selected by considering the intersection between the FLAIR lesion and the white matter of the Desikan–Killiany atlas. (**d**) FLAIR MRI lesion in PET space is used in the segmentation process in case of low-grade neoplasms. For high-grade neoplasms, this module employs an automatic thresholding to segment PET tumors. (**e**) After lesion delineation, the last module extracts static and dynamic parameters within the tumor, the reference, and the striatum region. All these measures are saved in CSV files for each subject.

The final version of the proposed pipeline is structured into 5 distinct modules, as illustrated in Figure 1: preprocessing, coregistration, region selection, tumor segmentation, and static and dynamic parameters extraction. The software code has been developed using Python 3.11 version (https://www.python.org), running in Centos 7, version 7.9.2009. Coregistration of different imaging modalities relies on Python code defined in Automated Pipeline for PET Imaging ANalysis (APPIAN) software [17], which requires all acquired images to be converted from Digital Imaging and Communications in Medicine (DICOM) to Neuroimaging Informatics Technology Initiative (NIfTI) format. This step was executed by utilizing MRIcroGL software version: v1.2.20220720b, available at the following link https://www.nitrc.org/projects/mricrogl/ visited 1 March 2023, which employs the dcm2nii function [18]. Additionally, the input data repository has been systematically organized following the Brain Imaging Data Structure (BIDS) standard [19]. For each subject, it is imperative to create a folder specific to the imaging modality, e.g., ‘anat’ for MRI, where both the NIfTI and the associated JSON file are placed. The pipeline is designed to delineate tumor volume with minimal manual intervention, applicable to both static and dynamic PET volumes. It employs an automatic thresholding algorithm to segment the [^18^F]F-DOPA PET tumor. This method directly relying on [^18^F]F-DOPA PET scan only works in case of neoplasms with both T/S and T/N ratios exceeding 1 in the two VOIs considered. Conversely, to include neoplasms with diminished [^18^F]F-DOPA uptake, the pipeline integrates the lesion drawn from FLAIR MRI using a region-growing algorithm implemented in MRIcroGL.

### 2.4. Preprocessing

#### 2.4.1. FLAIR MRI Segmentation

**Figure A1:**
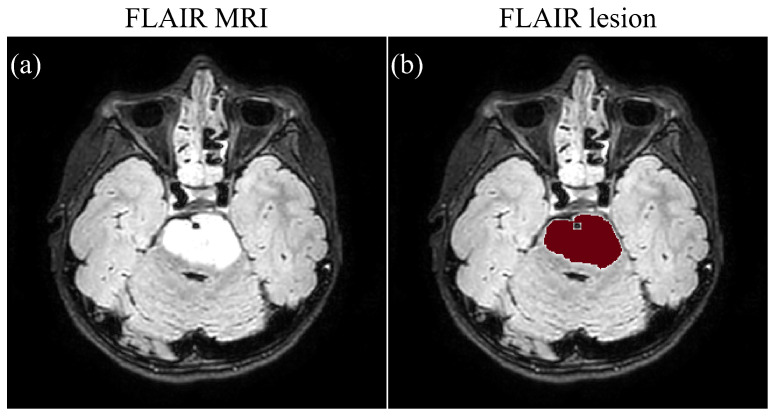
We show an axial slice of MRI FLAIR of an exemplary subject affected by a diffuse intrinsic pontine glioma. (**a**) The lesion appears hyperintense. (**b**) We report the superimposition of the binary mask obtained with the semi-automatic growing region method of MRIcroGL.

FLAIR MRI tumor volume was outlined using the advanced drawing tool of MRIcroGL. This tool operates in a semi-automatic mode and employs a region-growing segmentation: the central portion of the lesions was chosen as a seeding point for the algorithm. The resultant VOI can be refined by adjusting two parameters: the radius of the VOI and the difference from the initial point. The binary volumetric images corresponding to the tumor volumes were incorporated into the pipeline as NIfTI files. In addition, the obtained VOIs were processed with a closing operator to enhance the homogeneity and uniformity of the mask. An example of this step is reported in Figure A1.

#### 2.4.2. Skull Stripping

**Figure A2:**
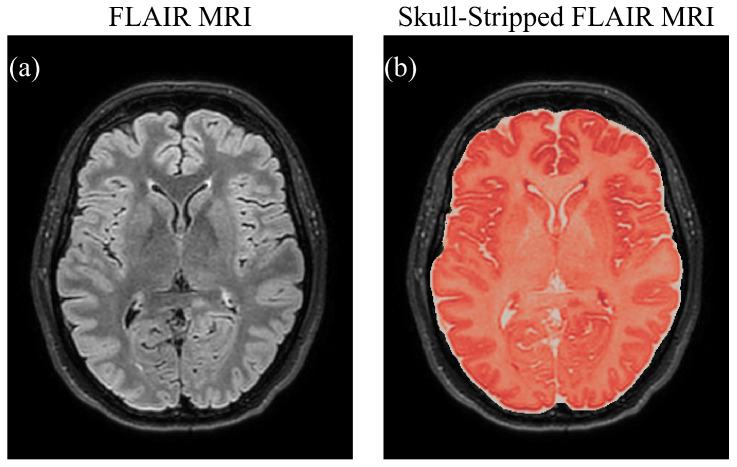
We report an axial slice of FLAIR MRI of an exemplary subject (**a**) original structural image. (**b**) Skull stripped MRI obtained using FSL BET tool.

Visual inspection of the static [^18^F]F-DOPA PET images revealed a significant uptake signal in non-encephalic regions, particularly in the skull. To enhance the accuracy of the analysis, we performed skull stripping on FLAIR MRI, using the BET tool, developed by the Oxford Centre for Functional MRI of the Brain (FMRIB), located at the University of Oxford, Oxford, United Kingdom within FSL [20]. The resulting skull-stripped images were then employed as a mask to isolate the brain, as shown in Figure A2.

#### 2.4.3. Erosion of Reference Region

**Figure A3:**
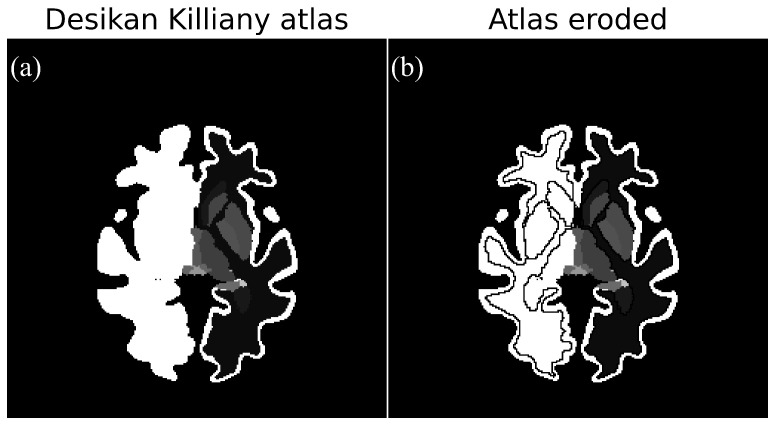
In this figure we show the comparison between the original Desikan–Killiany atlas and the one obtained after a cycle of erosion. (**a**) It is possible to see how the voxels of gray matter, white matter, and subcortical structures are not separated. (**b**) After erosion of the white matter, of both hemispheres, all structures become distinguishable. We chose the eroded atlas to reduce the partial volume effect in subsequent pipeline steps.

The Desikan–Killiany atlas (DKA) [21] was applied to automate the identification of white matter and corpus striatum regions from both hemispheres. The white matter volumes, designated as reference regions, were eroded to mitigate the spillover effect, which might arise around regions with intensive tracer uptake (Figure A3). The erosion of white matter is specifically conceived to separate the signal of the reference region from adjacent encephalic structures.

### 2.5. Coregistration

In this module, our code coregisters Montreal Neurological Institute [22] and DKA atlases and all FLAIR MRI volumes into the PET coordinate space. The alignment and transformation steps are based on the APPIAN software [17]. The alignment procedure consists of two steps: (i) the MRI volume is aligned with the PET, and (ii) the Montreal Neurological Institute atlas is aligned with the MRI. To perform this first step, the align function was defined using the registration function provided by Python’s ANTspy library [23]. The PET volume, established as the reference image, and the MRI volume, which is the source image, were used as inputs for this function. Considering that both the source and the reference images refer to the same subject, a rigid body (6 parameter) linear transformation is applied. The output of the align function provides the transformation to move from the MRI system to the PET coordinate space. MNI atlas was aligned to the MRI using a non-linear transformation with ANTs. For the transformation step, the MNI to PET coordinate transformations were applied to the DKA and tumor VOI to warp them into PET coordinate space using nearest neighbor interpolation.

### 2.6. Region Selection

This study focuses on individuals affected by hemispheric or subtentorial tumors. For hemispheric tumors, the white matter of the opposite hemisphere was employed as the reference region, and the selection of the reference region was verified by overlaying the tumor outline on key regions such as the white matter, thalamus, and pons. The striatum was defined by combining specific areas for each hemisphere based on the look-up table. When dealing with subtentorial tumors, the left hemisphere was consistently employed as the reference.

### 2.7. Tumor Segmentation

Before identifying lesions, the defined pipeline removes the skull signal from the [^18^F]-FDOPA PET using a brain mask obtained from the skull-stripped FLAIR. We implemented a thresholding algorithm that automatically identifies tumor lesions within the [^18^F]-FDOPA PET volume. To obtain the maximum radiotracer uptake value of the reference region, the entire white matter in the hemisphere opposite the lesion was considered. The semioval center was not included because the DKA atlas does not encompass this region and we chose not to manually add this VOI to the atlas in order to minimize manual intervention. The semioval center was not considered because the DKA atlas does not encompass this region and we chose not to manually add this VOI to the atlas in order to minimize manual intervention. Furthermore, considering a large reference region, such as the entire white matter, provides greater reliability of its maximum value and the extracted parameters:

(1) $$SUVr_{max}=\frac{PET_{3D}}{max_{N}}$$

(2) $$SUVr_{max}>1$$

The SUVr volume represents a map of the radiotracer uptake normalized to the maximum concentration level within the white matter of the hemisphere opposite to the lesion site. The voxels with concentration values above the maximum observed in the reference region define the tumor volume. Once the PET tumor volume was defined, the T/S and T/N ratios were calculated for both the PET and FLAIR tumor VOI. The automatically derived volume was used in subsequent pipeline modules only if all parameters had values greater than 1; otherwise, the FLAIR-drawn VOI was used.

### 2.8. Enhancement of Tumor Segmentation

In static PET images derived from dynamic scans, a high uptake signal from the superior sagittal sinus is frequently observed. This was also our case within the tumor mask. To address this issue, we devised a two-step approach, as follows:

1. A probability map representing the sinus sagittalis was obtained by performing a weighted sum of the initial two frames of the 4D PET scan. The first two frames were employed because the sagittal sinus signal decays in the following frames. Normalizing this volume to its maximum yields a pseudo-probability map.
2. Next, the tumor mask was refined by removing small connected components and integrating it with the FLAIR tumor using logical OR operation. A Gaussian filter, with FWHM equal to 5, was applied to generate a probability distance map.

**Figure 2:**
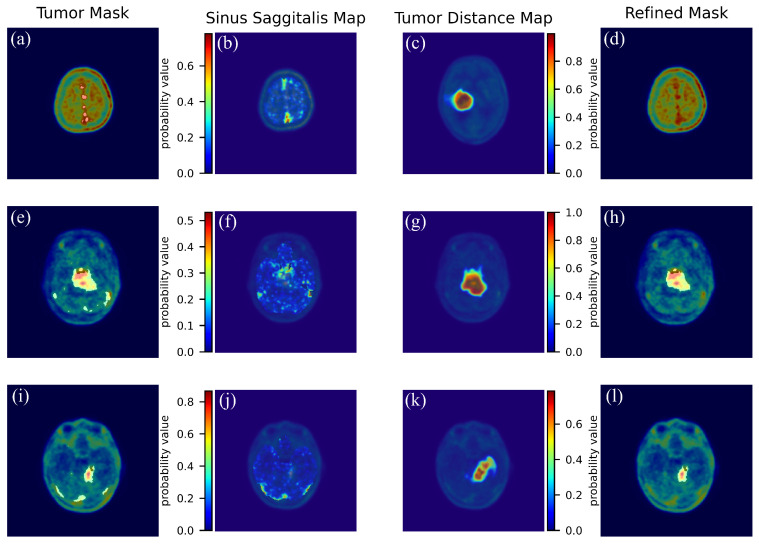
Refinement step for automatically segmented lesions. We present exemplary cases of subjects with: a diffuse midline glioma (DMG), H3 K27-altered involving the right thalamus (**a**–**d**), a DIPG (**e**–**h**), and a DMG, H3 K27-altered of the left diencephalic–mesencephalic junction (**i**–**l**). The tumor masks in the first column encompass the superior sagittal sinus. Probability maps consistently reveal elevated values within this structure. The third column displays the lesion’s distance map. In the last column, refined tumor regions are shown after excluding the sinus structure. This refinement eliminates artifacts and provides a clearer representation of the tumor.

Subsequently, probability values were extracted within the original tumor mask for both maps. These values served as inputs for training a K-means algorithm to discern between tumor and sinus labels [24]. Following label assignment, Support Vector Machine (SVM) was employed as a classifier to enhance the accuracy of tumor–sinus classification [25]. This approach aligns with recent advancements in clustering functional magnetic resonance imaging (fMRI) time series, which have shown promise in glioblastoma characterization by enhancing the identification of tumor regions [26]. Both algorithms are implemented in the scikit-learn Python library [27]. This step was executed only for the lesions with T/S and T/N both > 1, an example of this step is reported in Figure 2.

### 2.9. Static Parameters Extraction

In DKA, the corpus striatum is defined by the aggregation of three different VOIs: caudate, putamen, and pallidum. Our pipeline assembles the striatal volume before static parameters analysis with OR operation. The maximum values within (i) the tumor region, (ii) the corpus striatum, and (iii) white matter are used for calculating the T/S and the T/N:

(3) $$\frac{T}{S}=\frac{Tumor_{max}}{Striatum_{max}}$$

(4) $$\frac{T}{N}=\frac{Tumor_{max}}{Reference_{max}}$$

### 2.10. Dynamic Parameters Extraction

**Figure 3:**
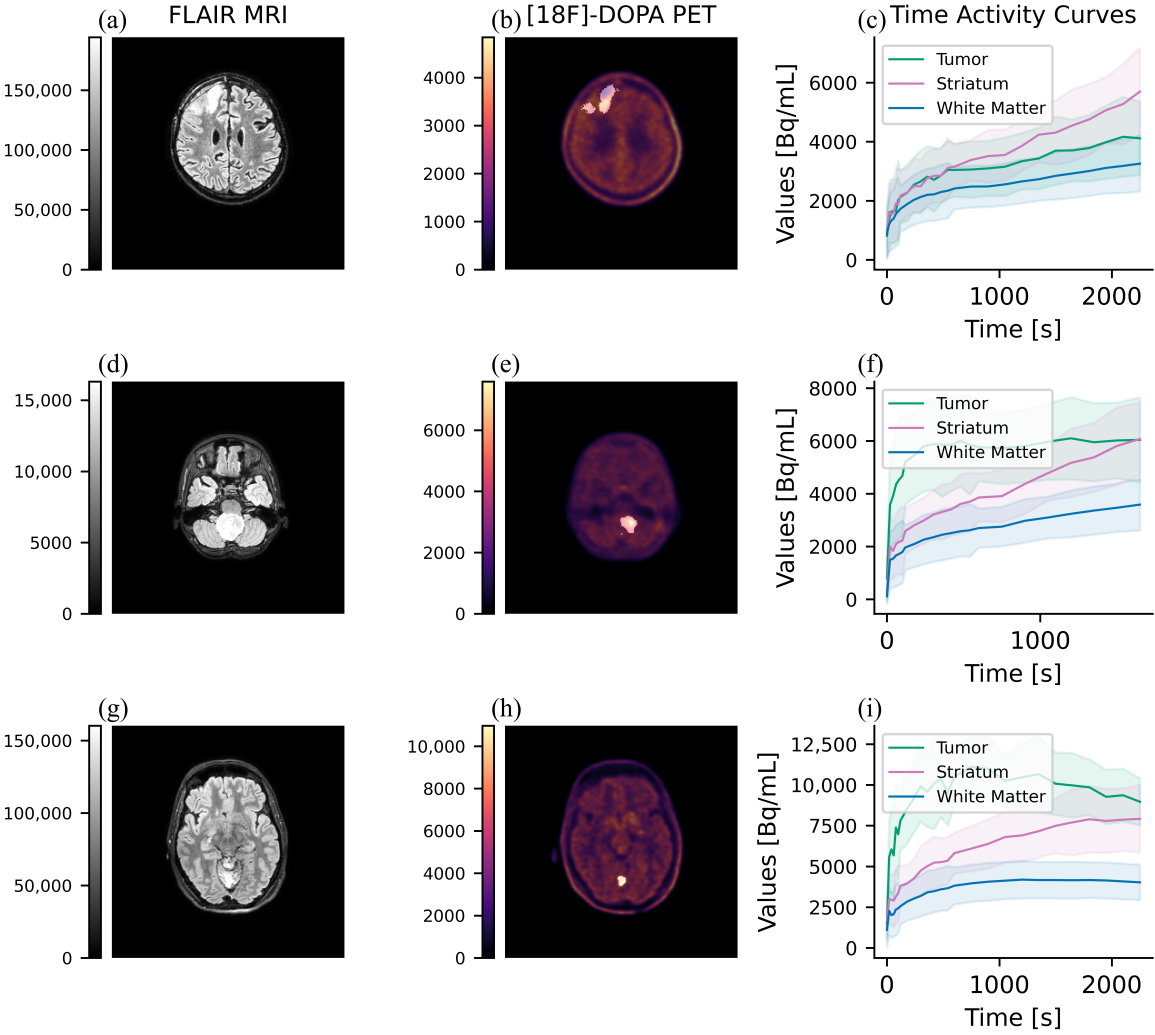
Time Activity Curves of tumor lesions. We report three exemplary subjects affected by: a Diffuse astrocytoma, NOS (WHO grade 2) of the right frontal lobe (**a**–**c**), a DMG H3 H27-altered (WHO grade 4) of the medulla (**d**–**f**), and a Pilocytic astrocytoma (WHO grade 1) of the cerebellum (**g**–**i**). In the first column, we report the FLAIR MRI. In the second column, we show the [^18^F]F-DOPA PET with superimposition of the lesion. In the third column, we report the Time Activity Curves showing three different patterns: (**c**): an accumulation pattern. (**f**): a plateau pattern (**i**): a downward pattern.

The dynamic parameters were calculated following the methodology outlined in reference [12]. The same VOI defined in the tumor segmentation step was used to calculate the dynamic indices. The 4D [^18^F]-FDOPA PET scans were used as input to generate time-activity curves (TAC), which were recorded in a CSV file for each subject. The TAC was calculated by averaging the uptake values within a specific VOI for each frame of the dynamic scan; the standard deviation is also reported in the CSV file. The dynamic parameters included Time-To-Peak (TTP), tumor slope, striatum slope, and the Dynamic Slope Ratio (DSR) [28]. TTP represents the time point at which the maximum uptake of TAC is reached. The two slopes were calculated via linear regression of the mean values, within a specific VOI, during the interval between the second minute and the end of the acquisition. DSR is represented as a normalization of the tumor slope to the striatum slope. The proposed semi-automatic method overcomes the limitations of manual tumor segmentation techniques in standard clinical practice. In Figure 3, we report exemplary cases of the 3 uptake pattern.

### 2.11. Intra-Tumor Heterogeneity Analysis

We analyzed voxel-level TAC within the tumor volumes to identify sub-regions based on radionuclide uptake. Three regions were hypothesized at the tumor level, demarcated by the following three intervals:

(5) $$Uptake_{maximum}=PET_{vol}>mean_{tumore}+2std_{tumor}$$

(6) $$\begin{array}{cc} Uptake_{mean}= & \;mean_{tumor}<PET_{vol}\\ \; & <mean_{tumor}+2std_{tumor} \end{array}$$

(7) $$\begin{array}{cc} Uptake_{minimum}= & \;mean_{tumor}-2std_{tumor}<PET_{vol}\\ \; & <mean_{tumor} \end{array}$$

**Figure 4:**
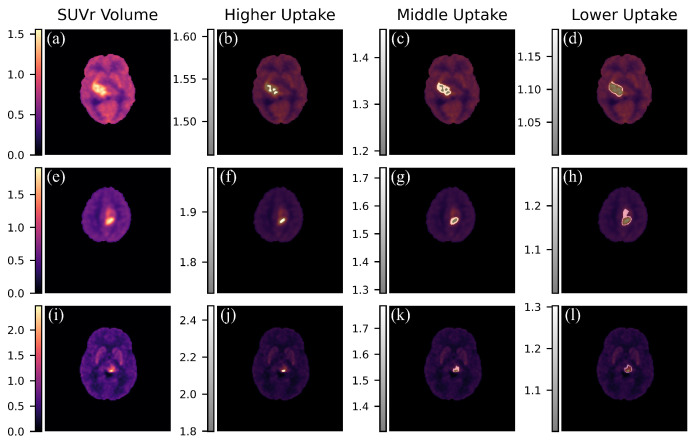
Visualization of tumor sub-volumes. (**a**–**d**): A Patient with a DMG H3 K27-altered (WHO grade 4) of the right thalamus. The first column depicts the [^18^F]F-DOPA PET SUVr volume. The following columns show the three sub-tumor masks. (**e**–**h**): A subject with a diffuse pediatric-type high-grade glioma, H3-wildtype, and IDH-wildtype (WHO grade 4) of the left paracentral lobule; in the following columns, we show the SUVr volume and the three masks. (**i**–**l**): Finally, we report a patient with a DMG H3 K27-altered (WHO grade 4) of the left thalamus, as shown in (**i**).

These regions were included alongside the DKA atlas as new labels, as shown in Figure 4. Finally, TACs were calculated to assess a possible differentiation in the uptake pattern of the three volumes considered. This step represents a first attempt to visualize different areas of hyperactivity, which can be crucial for radiotherapy planning.

### 2.12. Manual Analysis

To validate our pipeline, static and dynamic indices were compared between the proposed extraction method and the conventional clinical workflow. The PET Volume Computer-Assisted Reading (VCAR) [29] tool was used on the GE HealthCare AW, located in Waukesha, Wisconsin, United States. server to analyze static indices. To calculate T/S ratios, the maximum value expressed in Bq/ml was calculated for both tumor and striatum using a growing region algorithm with the maximum value as the seeding point to establish these VOIs. For T/N ratios, the reference region was determined using a sphere in the contralateral centrum semiovale. The radius of the sphere was adjusted for each subject to include both white and gray matter. TACs were extracted with the 3D PET viewer on the AW server with the PET 4D tool, allowing for visualization and extraction of the TAC. The tumor lesion was manually defined using a growing region method in the final frame of acquisition, due to higher anatomical detail. This VOI was then projected to all other frames, and the TAC was saved as a CSV file for further analysis. A correlation analysis of the T/S ratios extracted by both the manual analysis and our algorithm was performed to assess reliability with the measurements reported in the clinical records, which were considered ground truth. This analysis specifically focused on the T/S ratio because it is the most reported metric and was based on a subset of 68 subjects, excluding those with negative PET scan results.

## 3. Results

### 3.1. Comparison Between Automatic and Manual Static Indexes

**Figure 5:**
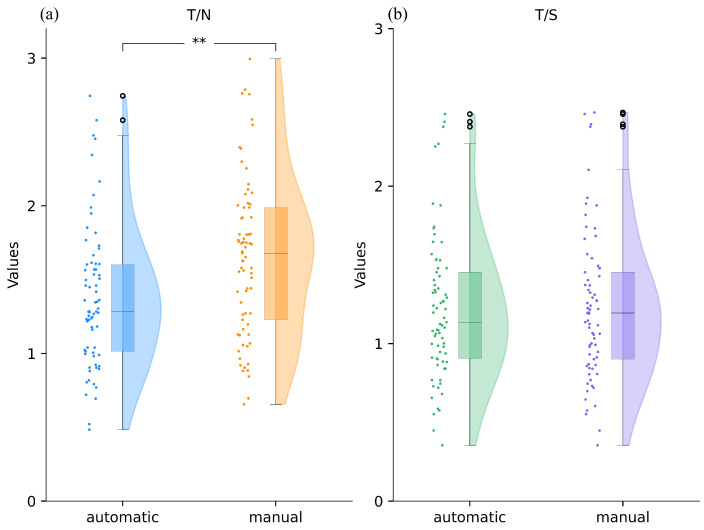
Raincloud plots depicting the distribution of T/N and T/S, computed with the proposed pipeline and with the manual method. Black dots refer to outliers. Panel (**a**) displays the distribution of the automatic T/N alongside the manual T/N index. These two indices have shown significant differences, as indicated by the ‘**’ in the figure, which denotes a *p*-value much less than 0.05. Panel (**b**) depicts the automatic T/S alongside the manual T/S index.

During the study period, 89 pediatric patients with brain gliomas who had undergone [^18^F]F-DOPA PET were evaluated. Out of the initial population, 73 subjects (37 females) were included in our study based on the selection criteria. Sixteen subjects were excluded due to the following reasons: ten subjects had a low resolution within the FLAIR MRI and we could not use it for coregistration; one subject, whose FLAIR was acquired post-surgery, was excluded because it showed signal abnormalities not compatible with the coregistration process; the remaining were excluded due to problems with the PET acquisition (two dynamic PET images had no signal within the volume, two subjects did not undergo PET acquisition due to health issues, and one dynamic PET had a different field of view to its static image). Static PET was available for all subjects, whereas dynamic PET scans were performed in 32. Our pipeline was employed within a cohort of 73 subjects and tested the performance of our algorithm by comparing the results with indexes and curves obtained manually. The manual and semi-automatic indices do not follow a normal distribution, as confirmed by the Shapiro–Wilk test. The two extraction methods were compared using the Wilcoxon test. For the T/S there are no statistical differences (*p* = 0.357), as shown in Figure 5b. Conversely, there is a significant difference for T/N (*p* < 0.005). As we are considering two metrics, the Bonferroni correction is applied. For T/S there are no statistical differences (*p* = 0.713), contrary to T/N (*p* < 0.005). Cohen’s D effect size and the confidence interval (CI) were calculated for the two static parameters. For T/S a D = 0.026 and CI = [−0.037, 0.014] were obtained. For T/N, however, the effect size was 0.55 and the CI = [−0.32, −0.23]. Cohen’s D was calculated according to [30].

### 3.2. Comparison Between Automatic and Manual Dynamic Indexes

**Figure 6:**
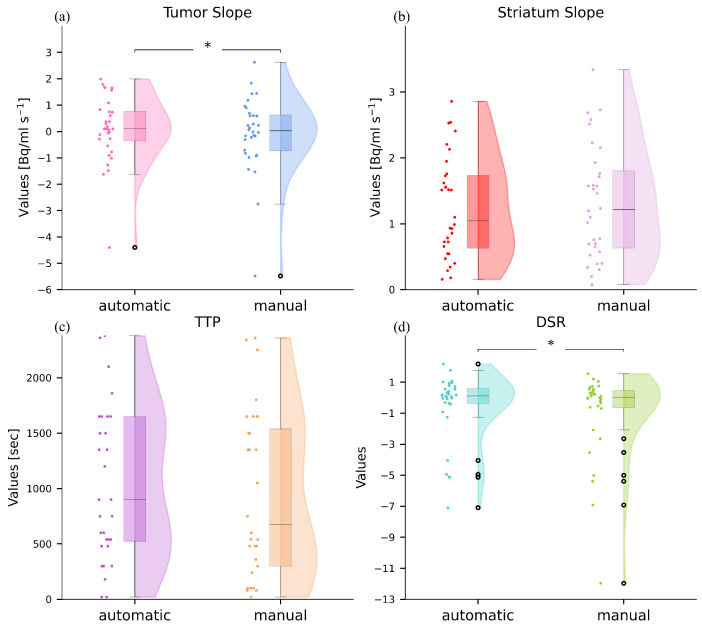
Raincloud plots depicting the distribution of the slopes, both for the tumor and striatum, the time-to-peak and the dynamic slope ratio derived from regions compared between the manual and the semi-automatic method. Black dots refer to outliers. Panel (**a**) displays the distribution of the semi-automatic tumor slope alongside the manual index, proposed pipeline and with the manual method. Black dots refer to outliers. Panel (**a**) displays the distribution of the automatic T/N alongside the manual T/N index. These two indices have shown significant differences, as indicated by the ‘*’ in the figure, which denotes a *p*-value much less than 0.05. Panel (**b**) depicts the semi-automatic striatum slope alongside the manual index. In (**c**), we display the distribution of the time-to-peak obtained using the two methods. In (**d**) we reported the semi-automatic and manual distributions of the dynamic slope ratio.

The dynamic indexes of 32 subjects were compared between the two methods. One subject was excluded because the dynamic PET contained noise due to [^18^F]F-DOPA administration error; a second subject was excluded because the tumor presented two dislocated lesions. All the dynamic indexes do not follow a normal distribution, as demonstrated by the Shapiro–Wilk test, so the Wilcoxon test was applied. No significant differences were found between the striatum slopes for the two methods (*p* = 0.369, *p* = 0.9 after Bonferroni correction) and the TTP (*p* = 0.167, *p* = 0.67 after Bonferroni correction). However, significant differences were observed between the tumor slopes and the DSR (*p* < 0.05, *p* = 0.09 after Bonferroni correction), as depicted in Figure 6a. Cohen’s D effect size and the CI were calculated for the dynamic parameters. For TTP, the effect size was D = 0.18 with a CI of [−33.12, 288.4]. The tumor slope yielded an effect size of D = 0.14 and a CI of [0.043, 0.34]. The striatum slope had an effect size of D = 0.088 with a CI of [−0.17, 0.017]. Finally, for DSR, the effect size was D = 0.17 and the CI was [0.094, 0.81].

### 3.3. Agreement Between Ground Truth, Manual and Automatic Static Indexes

A subset of 68 subjects was employed in this analysis, excluding those with negative PET scans due to the absence of T/S ratios in their reports. To evaluate the reliability of the manual analysis and our tool in segmenting the tumor region, an intra-class correlation (ICC) analysis was performed against the ground truth from the clinical reports. First, the ICC (type 3) were calculated between the T/S ratios from the reports and those obtained through manual analysis, resulting in an ICC of 0.91. Then, the average T/S ratio was obtained from manual analysis and clinical reports. The ICC between the T/S obtained by our tool and this averaged T/S ratio were calculated, achieving an ICC of 0.94. ICC analysis was performed with the function implemented in Python [31].

## 4. Discussion

There are several open-source software that allow for automated analysis of PET images. In 2018 Funck et al. [17] introduced APPIAN, a comprehensive tool designed to automate the analysis of PET scans. This pipeline encompasses critical stages, including coregistration with structural MRI images, partial volume correction, and quantification. Notably, APPIAN is developed using Nipype, making it open-source and compatible with various programs executable in a Bash shell environment. Its modular architecture facilitates easy expansion and customization, empowering users to integrate new algorithms or entirely novel analyses into the workflow. In 2023, Nordio et al. [32], implemented a pipeline to perform kinetic analyses of [^18^F]-DOPA PET. The main steps can be summarized as follows: image motion correction, coregistration with stereotactic atlases, and quantification. To the best of our knowledge, these existing tools primarily facilitate quantitative studies of predefined regions already mapped in an atlas. However, to apply these frameworks effectively in the clinical routine examination of brain tumors, physicians would still need to delineate the tumor region manually. Consequently, this approach does not completely address the need for automation in nuclear medicine analysis. Peira et al. [33], in 2023, defined an automatic approach for the extraction of static parameters from [^18^F]F-DOPA PET in pediatric diffuse gliomas. The analysis follows the clinical workflow defined in [34]. This work represents a first attempt at the implementation of an automatic computer-aided tool to assist clinicians in the extraction of semi-quantitative parameters. This software is structured in three modules: (i) spatial normalization of the PET volume to the MNI atlas, (ii) identification of the non-affected brain hemispheres, and (iii) extraction of the standardized uptake value ratio (SUVr) volume and static parameters. The source code is defined in MatLab, which limits the availability of the code since it is not an open-source environment. Furthermore, this code is not structured into a pipeline, as the coregistration was implemented with Insight Toolkit (ITK, version 4.12) and all subsequent steps are implemented in MatLab. Lastly, this software does not compute dynamic parameters. With our work we present a tool designed to assist nuclear medicine clinicians in assessing pediatric brain gliomas by automatizing the extraction of both static and dynamic parameters. This tool was tested on a cohort of 73 pediatric patients, showcasing a variety of ages and tumor aggressiveness. In our study, the indexes obtained automatically by the pipeline were compared against those derived from the manual method performed with PET VCAR tools. Our software is open-source, written entirely in Python, and has a modular and extensible architecture that allows users to add new elements to the analysis. The proposed pipeline offers several significant advantages over manual analysis. Firstly, it mitigates individual interpretation errors and provides a reliable aid in clinical decision-making. Additionally, it ensures faster processing times and offers an accessible framework for clinicians with varying experience levels. By employing a hybrid approach for tumor segmentation, our software is also effective for lesions with low [^18^F]F-DOPA uptake. This approach mitigates potential uncertainties associated with automated segmentation in such instances. In the literature, the centrum semiovale, comprising both white and gray matter, is commonly used as the reference region. However, in our study, the entire contralateral white matter to the tumor lesion was identified as reference. Indeed, we believe that considering a larger region enhances the reliability of the extracted maximum value. The reason behind observing a significant difference between semi-automatic and manual T/N ratios is precisely attributable to the different reference regions employed (*p* < 0.005). Nevertheless, the maximum uptake value of N is used for tumor segmentation, and consequently, the maximum lesion absorption value is employed to calculate T/S. For this second index, we have demonstrated that our software achieves good reproducibility compared to the manual method and the ground truth. The Wilcoxon test was employed to statistically analyze these differences. For the T/S ratio, no statistically relevant differences were found between the semi-automatic and manual approaches (*p* = 0.357, *p* = 0.713, after Bonferroni correction). To further quantify these findings Cohen’s D effect size and CI were calculated. For the T/S ratio, Cohen’s D was 0.026 with a confidence interval of [−0.037, 0.014]. This negligible effect size and the narrow CI strongly support the conclusion that there are minimal differences between the semi-automatic and manual T/S measurements. The consistency of T/S across methods reinforces the reliability of the proposed software in accurately replicating the manual analysis for this parameter. These results are particularly important for two reasons: (i) it indicates that the DKA atlas is accurately coregistered with the PET images, and (ii) it ensures consistent identification of the tumor VOI. Conversely, for T/N, the effect size was larger, with Cohen’s D equal to 0.55 and a confidence interval of [−0.32, −0.23]. These results indicate a pronounced difference between the two methods for T/N, further highlighting the impact of reference region selection. One of the most innovative parts of our work is the automatic extraction of TAC and dynamic parameters from tumor lesions, which significantly leverages clinical workflow. It is also one of the first projects to automate the extraction of dynamic parameters. Typically, calculating dynamic parameters is onerous and heavily reliant on the user’s experience in lesion delineation. The proposed software employs the segmented tumor lesion from the static PET scan as the VOI for curve extraction. In contrast, physicians usually delineate the lesion in the last frame of the dynamic acquisition and back-project it onto previous frames. This approach is challenging due to the reduced anatomical information of dynamic PET scans, complicating the segmentation process. Although PET VCAR tools allow a growing region algorithm to accelerate segmentation, the defined volume often requires refinement to minimize artifacts from adjacent regions in the TAC calculation. Moreover, this method is only effective for lesions with an absorption of the radiopharmaceutical higher than cerebral parenchyma. For tumors with low uptake, it is necessary to utilize information derived from structural MRI. Our method accelerates these steps by delineating the FLAIR lesion as a pre-processing step and performing artifact removal from the tumor region as an automatic refinement process. To minimize the need for manual post-processing, a tumor VOI refinement step was implemented to remove all non-tumor voxels from the lesion mask. The statistical analysis of the dynamic parameters revealed varying outcomes between the manually and automatically calculated indices. For the striatum slopes, no significant difference was found between the two methods (*p* = 0.369, *p* = 0.9 after Bonferroni correction), and similarly, the TTP did not show a significant difference (*p* = 0.167, *p* = 0.67 after Bonferroni correction). However, for the tumor slopes and the DSR, significant differences were observed (*p* < 0.05). The effect size for TTP was small, with a Cohen’s D value of 0.18 and a wide CI of [−33.12, 288.4]. The tumor slope had a similarly low effect size (D = 0.14) and a CI of [0.043, 0.34]. The striatum slope yielded an even smaller effect size (D = 0.088) with a CI of [−0.17, 0.017]. Finally, for DSR, the effect size was D = 0.17, with a CI ranging from [0.094, 0.81]. These results suggest that, although there are some differences, the small effect sizes indicate that the variations between manual and automatic methods are likely not significant, except for tumor slope and DSR. Moreover, the wide CI of the TTP, despite the lack of significant differences, suggests the importance of better investigating the reliability of the tool in extracting dynamic parameters by increasing the cohort of patients with available dynamic scans.

## 5. Limitations

The principal constraints of this study can be summarized as follows: (i) the semi automatic approach deriving from the inclusion of cases with low uptake and (ii) the low number of subjects with dynamic PET availability. The initial aim was to define a fully automatic tool; however, during the development of the code, it became evident that it was not feasible to employ a thresholding algorithm for cases of tumors with a low radionuclide uptake. Moreover, the limited number of subjects in the Galliera Hospital database constrains the possibility of employing deep learning (DL) techniques for a fully automated analysis of [^18^F]F-DOPA PET imaging. The final software is therefore semi-automatic, as the FLAIR tumor lesion is delineated using the MRIcroGL software. The second limitation is related to the low number of subjects with the availability of dynamic PET scan. Indeed, this imaging modality was only introduced in the nuclear medicine department in 2018. The statistical results obtained on the dynamic parameters represent an initial and preliminary analysis. The small sample size for dynamic PET scans introduces challenges regarding the reliability and generalizability of the findings. Future studies with larger datasets will be necessary to confirm the preliminary trends observed. One last significant constraint is that the resolution of MRI data can affect the coregistration process. In fact, different subjects were excluded due to signal abnormalities within the FLAIR MRI due to low resolution or different protocols applied in the acquisition protocol.

## 6. Conclusions

Our work introduces a new framework to assist nuclear medicine physicians in analyzing [^18^F]F-DOPA PET imaging for pediatric brain gliomas. Our study showed that this framework reliably extracts static parameters across a large group of pediatric patients and automates the extraction of dynamic data. Traditionally, analyzing [^18^F]F-DOPA PET scans involves a lot of manual work, but our framework streamlines this process, providing more reliable and reproducible results. Additionally, it simplifies segmentation and extraction processes, paving the way for the broader adoption of dynamic metrics in the diagnosis and prognosis of brain gliomas. To assist clinicians in the prognostic process, this study focused on the extraction of fundamental parameters. In future works, we intend to investigate the correlation between these metrics and significant clinical outcomes, e.g., OS, PFS, and genetic mutations. Furthermore, the next target is to investigate the potential of radiomics analysis within this cohort, with a view to identifying which characteristics may prove more informative in terms of outcome. Additionally, a comparison between the developed tool and various existing platforms, such as APPIAN, will be conducted to evaluate processing times and examine the correlation of the extracted clinical metrics to better understand the reliability of the defined tool. 

## Figures and Tables

**Table 1:** Characteristics of the study cohort.

Variable	Categories	Subjects Included (%)
Sex	Female, *n* (%)	37 (50.6)
	Male, *n* (%)	36 (49.3)
Age on diagnosis, median	<12	35 (47.9)
	≥12	38 (52)
Classification of Tumors	Circumscribed astrocytic gliomas	6 (8.2)
	Glioneuronal and neuronal tumors	4 (5.4)
	Pediatric type diffuse low-grade gliomas	16 (21.9)
	Pediatric type diffuse high-grade gliomas	47 (64.3)

## Data Availability

The dataset supporting the conclusions of this article is not publicly available due to privacy restrictions of clinical data imposed by the Galliera Hospital’s administration but is available from the corresponding author on reasonable request. Code for our pipeline is available at https://github.com/MicheleMureddu/Pediatric_fdopa_pipeline. Current software has been designed and tested under Python 3.11.
